# Supplementary material for: Bisected graph matching improves automated pairing of bilaterally homologous neurons from connectomes
Source: Netw Neurosci. 2023 Jun 30;7(2):522–38. doi: 10.1162/netn_a_00287 (PMC10319359; doi:10.1162/netn_a_00287)
Supplement: Supplementary file 1 [file netn-7-2-522-s001.pdf]

Pedigo, B. D., Winding, M., Priebe, C. E. & Vogelsein, J. T. (2022). Supporting information for "Bisected graph matching improves automated pairing of bilaterally homologous neurons from connectomes." Network Neuroscience. *Advance publication*. https://doi.org/10.1162/netn_a_00287.

Journal: **NETWORK NEUROSCIENCE**

METHODS

Supplemental Information: Bisected graph matching improves automated pairing of bilaterally homologous neurons from connectomes

Benjamin D. Pedigo[1*], Michael Winding[2], Carey E. Priebe[2], and Joshua T. Vogelstein[1]

[1]Biomedical Engineering, Johns Hopkins University, Baltimore, USA

[2]Zoology, University of Cambridge, Cambridge, UK

[*]Corresponding author: bpedigo@jhu.edu

Keywords: Structural Connectome, Graph Matching, Network Alignment, Network Analysis, Homology, Bilateral Symmetry

SUPPLEMENT: UNDERSTANDING MATCHING FOR WEAKLY CORRELATED NETWORKS

We sought to understand why bisected graph matching can decrease matching accuracy when the correlation in the contralateral subgraphs is weak. To do so, we repeated the simulation from Matching simulated networks with a contralateral edge correlation of 0.1. Recall that for this value of the contralateral edge correlation, bisected graph matching achieves a matching accuracy of around 0.6, while for graph matching, accuracy is around 0.8. After simulating networks from this model, we computed the number of edge disagreements in the contralateral connections under four different permutations of the ”right hemisphere”: the true alignment used to generate the network, the permutation predicted by graph matching, the permutation predicted by bisected graph matching, and the permutation predicted by ”contralateral graph matching” (CGM, i.e., setting the ipsilateral networks to the zero matrix and running BGM).

For the parameters of the model used here, we observed that the true alignment of these networks had, on average, 26 edge disagreements (Supplemental Figure 1). Note that for unweighted networks, the

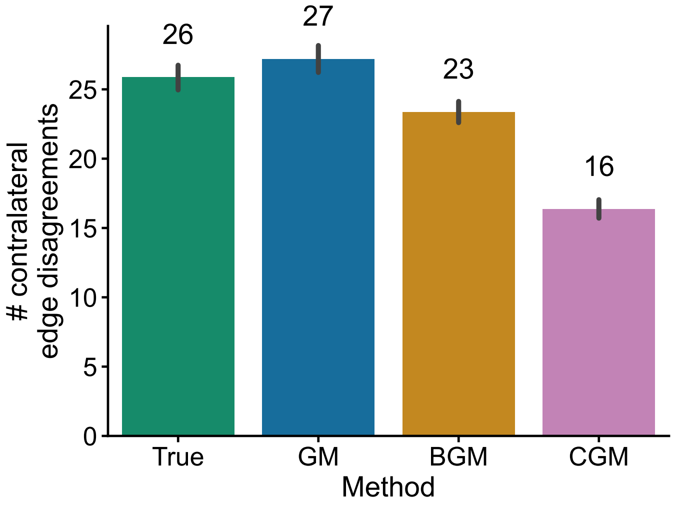

Supplemental Figure 1. Examination of the number of edge disagreements after matching for the same network model described in **??** with a contralateral edge correlation of 0.1. Bars show the mean number of edge disagreements over 1,000 runs from the true alignment and the permutations predicted by graph matching (GM), bisected graph matching (BGM), and contralateral graph matching which ignores the ipsilateral subgraphs (CGM). Note that BGM and CGM are finding permutations under which there are fewer edge disagreements than under the alignment the contralateral networks were sampled from—in other words, they are finding permutations under which the contralateral networks are more similar to each other. This phenomenon explains why BGM has lower matching accuracy relative to the true permutation in Figure 2 when the contralateral correlation is very weak.

graph matching objective functions are trying to minimize the number of edge disagreements between the respective subgraphs being matched. The permutation predicted by graph matching yielded 27 edge disagreements—it is unsurprising that this is close to the true number, as graph matching is often recovering the true alignment, as shown in Figure 2. However, we observed that bisected graph matching and contralateral graph matching yielded 23 and 16 edge disagreements on average, respectively. What should we make of the fact that there are actually *fewer* edge disagreements than under the permutation used to generate these subnetworks under the correlated Erdős-Rényi model?

This phenomenon was studied for correlated Erdős-Rényi networks in Lyzinski, Fishkind, and Priebe (2015), where the authors showed that if the correlation between the two networks is sufficiently weak, then the number of permutations which align the networks better than the true permutation goes to infinity as the networks grow. In other words, we can expect the performance of any graph matching algorithm to be poor (with respect to the true permutation) if the network correlations are weak. Given this phenomenon, (Fishkind et al., 2021) discussed how to assess these so-called "phantom alignments," proposing methods for assessing whether a matching is likely to have arisen by pure chance under some model of uncorrelated networks. While these kinds of methods could also be applied to the bisected graph matching setting, they still do not provide a method for inferring a better matching for these weakly correlated networks, so we did not explore them further here.

SUPPLEMENT: EFFECT OF NETWORK SIZE IN SIMULATED EXPERIMENTS

In an extension to the simulation described in Matching simulated networks we verified that the phenomenon shown in Figure 2 would hold over a range of network sizes. We used the same correlated Erdős-Rényi model described in Matching simulated networks, but varied the number of nodes in the simulated network. Specifically, we swept over a range of sizes which corresponded with the network sizes we studied for the real connectome datasets in Matching connectomes.

Supplemental Figure 2 shows matching accuracy for GM and BGM as a function of network size, for contralateral correlations of 0.2, 0.4, and 0.6. We observed that again, when the contralateral correlation is too small (0.2), BGM performs worse than GM. However, for even modest correlation (0.4, 0.6), BGM provides a large improvement over GM across the entire range of network sizes. In fact, the gap between BGM and GM grows as the networks get larger. For instance, for networks with 600 nodes per side and a

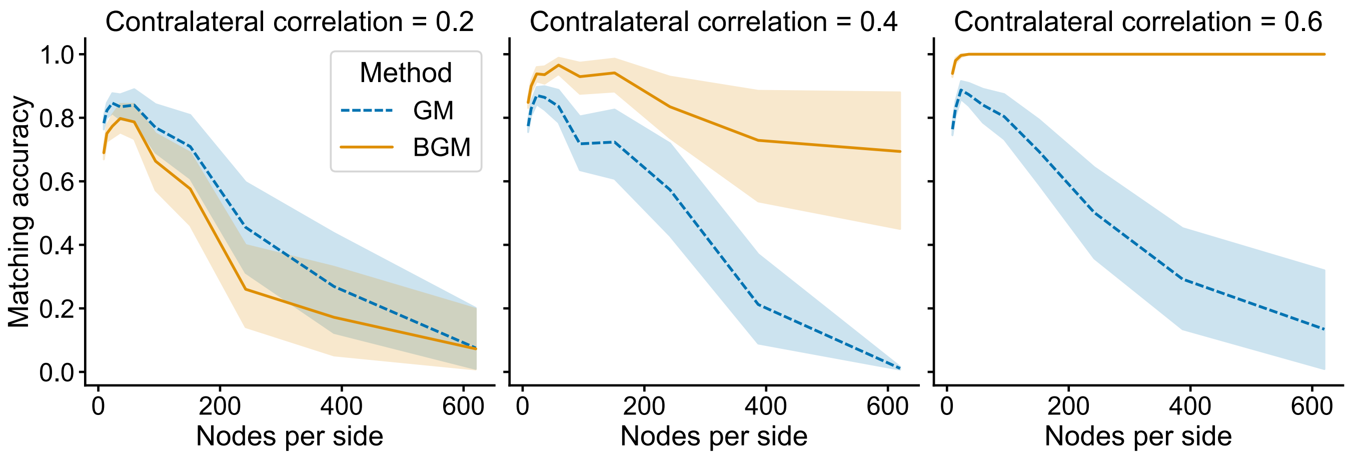

Supplemental Figure 2. Comparison of matching accuracy for graph matching (GM) and bisected graph matching (BGM) as a function of network sizes, shown for varying contralateral correlations. Simulated networks were constructed following the same correlated Erdos-Renyi model as in Matching simulated networks, except the number of nodes (n) for each network was varied. As in Figure 2, when the contralateral edge correlation is too weak (0.2), then including the contralateral subgraphs in the optimization (BGM) does not improve performance for any network size. However, if the correlation is stronger (0.4 or 0.6 for this simulation), then BGM provides a large increase in matching accuracy across a range of network sizes.

contralateral correlation of 0.6, GM is only matching with an accuracy of around 0.2, while BGM is matching perfectly.

SUPPLEMENT: MATCHING WITH SIMULATED UNPAIRED NEURONS

In many real connectome datasets, some subset of neurons may not have a homolog on the other side of the nervous system. Thus, we sought to understand the robustness of GM and BGM to the presence of these “unmatchable” nodes. To do so, we performed synthetic perturbations of the connectomes studied in Matching connectomes. For each dataset, we randomly selected some proportion (ranging from zero to 0.25) of neuron pairs for perturbation. For these pairs, we shuffled the edges to and from these neurons, removing any edge correlations with their respective partners on the other side of the nervous system. Note that because some of these edges will be to/from the set of neurons which are still matched, this process will also make it harder to match those neurons as well. Then, we applied the graph matching and bisected graph matching algorithms to these perturbed connectomes, and measured the matching

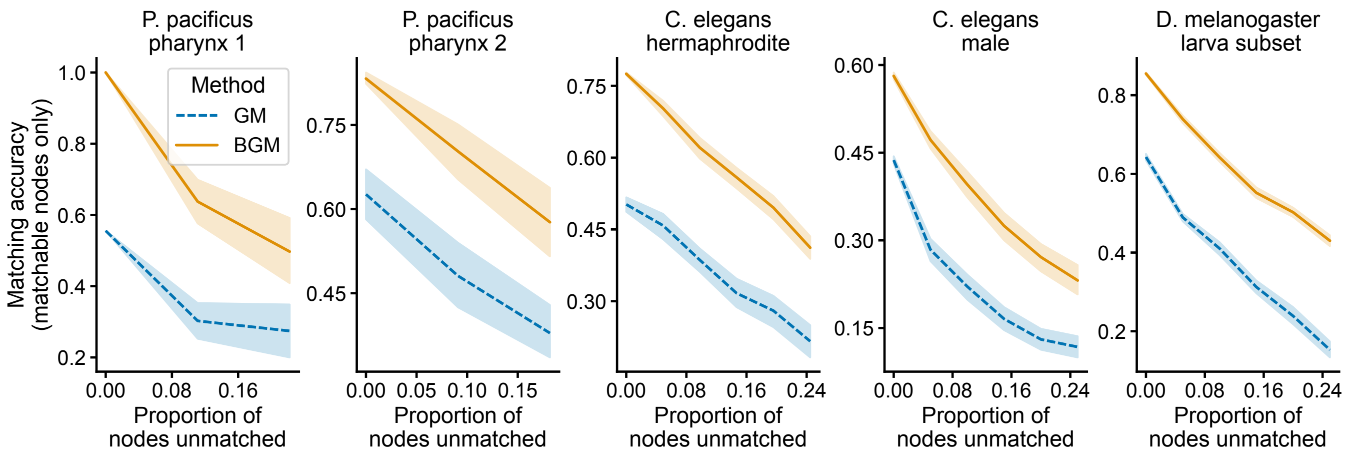

Supplemental Figure 3. Mean matching accuracy across 50 simulations for graph matching and bisected graph matching for perturbations of the connectomes studied in Supplement: matching with simulated unpaired neurons. For each connectome (panels), some proportion (x-axis) of neuron pairs had their incident edges shuffled, removing any edge correspondence for those pairs. For each dataset and proportion of neuron pairs perturbed, bisected graph matching showed a higher mean matching accuracy (y-axis) for the remaining matchable nodes.

accuracy for the unperturbed neurons. This process was repeated 50 times for each dataset and percentage of neurons to perturb.

Supplemental Figure 3 shows the matching accuracy for GM and BGM as a function of the proportion of pairs perturbed for all five connectome datasets. Unsurprisingly, unmatching some nodes by shuffling their incident edges does degrade the performance of both algorithms. However, we observed that in all cases, BGM had a higher matching accuracy than GM. Thus, BGM improves robustness in the presence of spurious nodes which have no true match on the other side of the nervous system.

SUPPLEMENT: DERIVATION OF BISECTED GRAPH MATCHING GRADIENT

Here, we show how the modified gradient (with respect to the permutation P) is computed for bisected graph matching. We start with the objective function for the bisected graph matching problem:

$$f(P) = \|A_{LL} - PA_{RR}P^T\|_F^2 + \|A_{LR}P^T - PA_{RL}\|_F^2$$

This can be written as the sum of a term for the ipsilateral subgraphs ($f_I(P)$) and one for the contralateral subgraphs ($f_C(P)$):

$$f(P) = f_I(P) + f_C(P)$$

Rewriting the first term:

$$f_I(P) = \|A_{LL} - PA_{RR}P^T\|_F^2 = tr((A_{LL} - PA_{RR}P^T)^T(A_{LL} - PA_{RR}P^T))$$

$$f_I(P) = \|A_{LL}\|_F^2 + \|A_{RR}\|_F^2 - 2tr(A_{LL}PA_{RR}^TP^T)$$

And likewise for the second:

$$f_C(P) = \|A_{LR}P^T - PA_{RL}\|_F^2 = tr((A_{LR}P^T - PA_{RL})^T(A_{LR}P^T - PA_{RL}))$$

$$f_C(P) = \|A_{LR}\|_F^2 + \|A_{RL}\|_F^2 - 2tr(A_{LR}^TPA_{RL}P)$$

We can drop terms unrelated to P and constants (since they will not affect the optimization) and returning to the full objective function we have:

$$f(P) = -tr(A_{LL}PA_{RR}^TP^T) - tr(A_{LR}^TPA_{RL}P)$$

Then, taking the derivative with respect to P for the first term:

$$\nabla f_I(P) = -A_{LL}PA_{RR}^T - A_{LL}^TPA_{RR}$$

Similarly for the second term:

$$\nabla f_C(P) = -A_{LR}P^TA_{RL}^T - A_{RL}^TP^TA_{LR}$$

So for the full objective function, the gradient is:

$$\nabla f(P) = -\left(A_{LL}PA_{RR}^T + A_{LL}^TPA_{RR} + A_{LR}P^TA_{RL}^T + A_{RL}^TP^TA_{LR}\right)$$

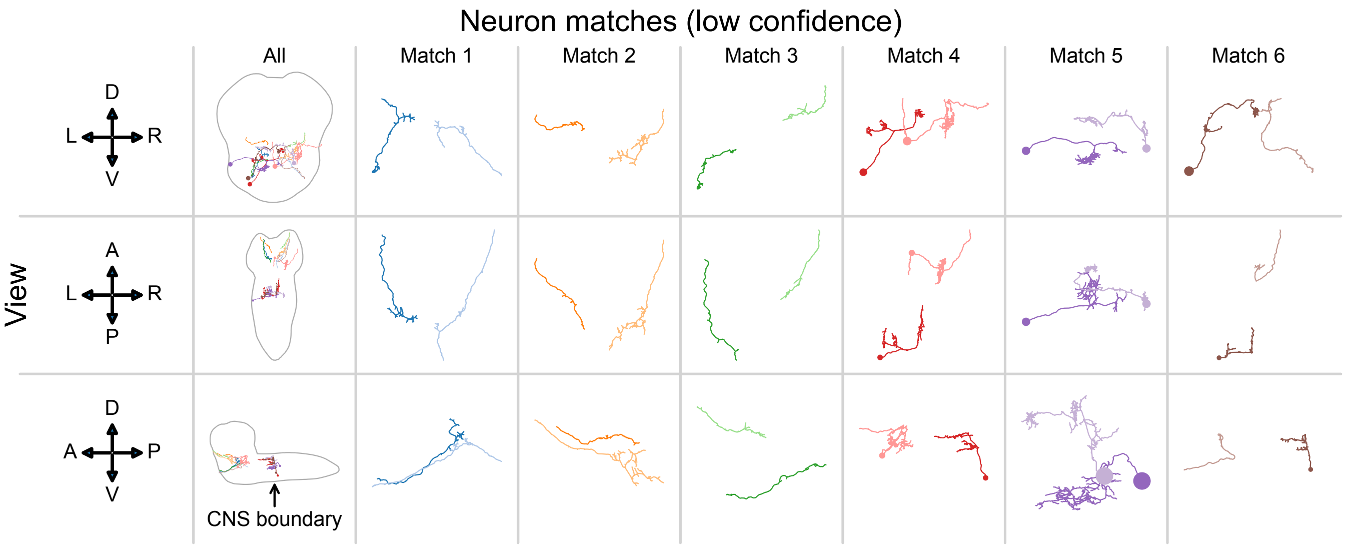

Supplemental Figure 4. Morphological comparison of low-confidence matched neurons in the *Drosophila* larva connectome subset (see Matching connectomes). Each column shows the best match for a left-hemisphere neuron which was frequently matched to different neurons across BGM initializations. Thus, these neurons often change partners over each initialization of the algorithm, suggesting that they are unlikely to be true bilateral homologs. Each row shows a different view of a matched pair of neurons (anatomical axes to the left show: D-dorsal, V-ventral, L-left, R-right, A-anterior, P-posterior). The morphology of these matched neurons appears different in most cases (compare to Figure 6), further supporting the idea that these matches represent implausible candidates for true bilateral homologs.

REFERENCES

Fishkind, D. E., Parker, F., Sawczuk, H., Meng, L., Bridgeford, E., Athreya, A., … Lyzinski, V. (2021). The phantom alignment strength conjecture: practical use of graph matching alignment strength to indicate a meaningful graph match. *Applied Network Science*, *6*(1). Retrieved from https://doi.org/10.1007/s41109-021-00398-z (Publisher: Springer International Publishing ISBN: 4110902100398) doi: 10.1007/s41109-021-00398-z

Lyzinski, V., Fishkind, D. E., & Priebe, C. E. (2015). Seeded graph matching for correlated Erdo"s-Rényi graphs. *Journal of Machine Learning Research*, *15*, 3513–3540.
